# Insights into the gut-liver axis: mechanisms and emerging therapies in hepatocellular carcinoma

**DOI:** 10.3389/fphar.2025.1595853

**Published:** 2025-05-19

**Authors:** Yongjian Hu, Mingming Gao, Jiajin Chenghuang, Rui Bao

**Affiliations:** ^1^ Division of Infectious Diseases in State Key Laboratory of Biotherapy, Center of Infectious Diseases, West China Hospital, Sichuan University, Chengdu, Sichuan, China; ^2^ HitGen Inc., Chengdu, Sichuan, China

**Keywords:** gut-liver axis, hepatocellular carcinoma, gut microbiota-derived metabolites, tumor microenvironment, therapeutic interventions, multi-omics analysis

## Abstract

The gut-liver axis is a multifaceted system where chemical and biological interactions between gut microbiota-derived metabolites and the liver significantly influence the development and progression of hepatocellular carcinoma Metabolites such as lipopolysaccharide (LPS), bile acids (BAs), and short-chain fatty acids (SCFAs) act as chemical mediators that modulate the tumor microenvironment through immune cell interactions and stromal activation, influencing tumor growth and metastasis. Changes in gut microbiota composition alter these signaling pathways, providing opportunities for therapeutic interventions. Strategies such as prebiotics, probiotics, and natural product-based small molecules have shown promise in modulating the gut-liver axis. Advanced multi-omics, chemical and bioinformatics tools, coupled with *in vitro* models like organoids, have unveiled intricate molecular interactions, offering insights into novel therapeutic targets. Future research should focus on delineating the pharmacological and immunological mechanisms within the gut-liver axis, developing personalized therapeutic strategies, and translating these findings into clinical applications.

## 1 Introduction

**FIGURE 1:**
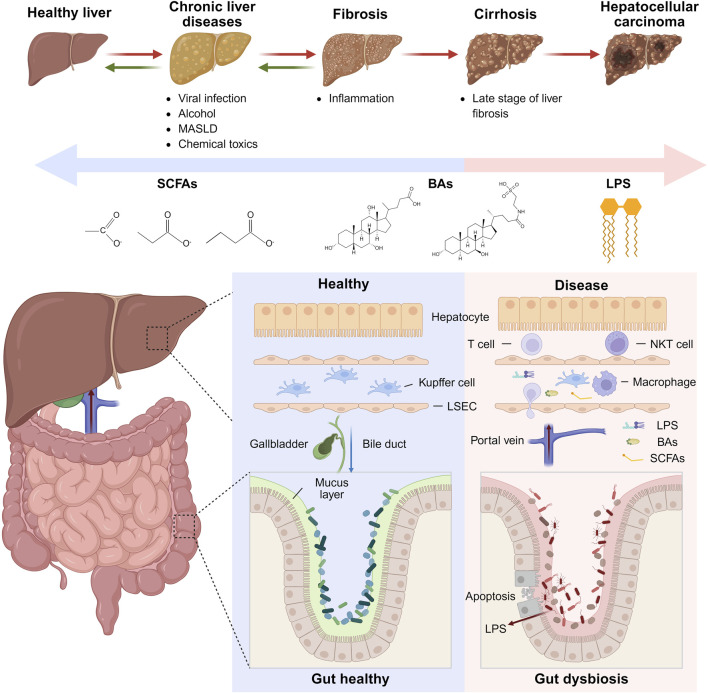
Interactions between gut microbiota and liver cancer progression. Upper Panel: The sequence of liver disease progression from a healthy liver through chronic liver diseases, fibrosis, cirrhosis, to HCC. Lower Panel: Comparison between healthy and diseased gut-liver states, emphasizing alterations in gut barrier integrity, immune cell infiltration in the liver, and the impact of harmful metabolites such as LPS on disease progression. Figure was created with BioRender.com.

The etiology and progression of HCC is a complex and multi-stage processes influenced by a variety of factors, including viral infections (e.g., Hepatitis B and C viruses), alcohol consumption, chemical substances (e.g., aflatoxins), and metabolic dysfunction-associated steatotic liver disease (MASLD) resulting from conditions such as obesity and diabetes. All these factors contribute to hepatocyte damage and inflammation. Hepatic fibrosis, primarily driven by the activation of hepatic stellate cells (HSCs), results in the deposition of fibrotic tissue and significant alterations in liver structure (Elpek, 2014). While fibrosis is a defensive response to inflammation or injury, chronic injury leads to excessive accumulation of extracellular matrix (ECM) components, thereby triggering hepatic fibrosis (Robinson et al., 2016). During this process, the liver ECM composition shifts from laminin and type IV collagen to interstitial collagen (Zadorozhna et al., 2020). As fibrosis intensifies and liver structure is disrupted, hepatocytes accumulate genetic mutations and epigenetic alterations, culminating in malignant transformation and HCC formation (Figure 1). Increasing evidence highlights the significant role of gut microbiota in human diseases, with interactions extending to extra-intestinal organs such as the liver (Singhvi et al., 2020). An inseparable bidirectional relationship exists between the liver and the gut, known as gut-liver axis, where bile and its metabolites are transported from the liver to the gut and microbial products are returned *via* the portal vein, can induce hepatic issues (Albillos et al., 2020). An imbalance in the gut-liver axis can lead to chronic inflammation and contribute to the progression of liver cancer (Arab et al., 2018). In the gut-liver axis, two barriers exist that play crucial roles in maintaining homeostasis and preventing the translocation of harmful substances.

The gut barrier consists of a single layer of columnar epithelial cells interconnected by tight junctions (Buckley and Turner, 2018). Goblet cells secrete a mucus layer that serves as the initial physical barrier (Zhang et al., 2022). The gut vascular barrier (GVB) is the innermost physical barrier, controlling the entry of microorganisms and luminal contents into the portal vein, thereby influencing hepatic immunity (Spadoni et al., 2015). Immune cells in the lamina propria, including intestinal macrophages and dendritic cells (DCs), phagocytose invading microorganisms and luminal contents (Luciani et al., 2022). Macrophages prevent pathogen-associated molecular patterns (PAMPs) such as LPS from entering the liver *via* the portal vein (Mowat et al., 2017). If PAMPs do enter the liver, they can affect the localization of immune cells within the liver (Bi et al., 2021). DCs guide antigens to mesenteric lymph nodes to activate adaptive immunity through resident Treg cells (Esterhazy et al., 2019; Cording et al., 2014). The liver barrier, similar to the gut barrier, comprises endothelial cells, mesenchymal cells, and immune cells, including macrophages, T cells, NK cells, and myeloid-derived suppressor cells (MDSCs), which filter and metabolize blood substances entering the liver *via* the portal vein (Liang et al., 2022). The liver hosts the largest population of tissue macrophages in the human body, primarily Kupffer cells (Roohani and Tacke, 2021). Gut microbiota and luminal contents reaching the liver are classified, recognized, and captured by liver immune cells (Gola et al., 2021). The gut-liver axis promotes HCC development through alterations in gut microbiota and microbial products, mucosal barrier damage, and GVB disruption (Mouries et al., 2019; Maccioni et al., 2020; Sorribas et al., 2019). Intestinal microbial imbalance and impaired barrier function allow harmful microbial products to enter the liver *via* the portal vein, triggering inflammation and exacerbating liver disease. Conversely, cirrhosis and portal hypertension alter the gut microbiome composition, facilitating microbial translocation to the liver, altering the hepatic immune environment, intensifying inflammation, and potentially inducing liver cancer.

This review aims to summarize recent advancements in understanding the mechanisms underlying the gut-liver axis in HCC, with a particular focus on the roles of key microbial metabolites such as LPS, BAs, and SCFAs. We will also discuss recent advancements in therapeutic strategies targeting the gut microbiota, including fecal microbiota transplantation (FMT), phage therapy, probiotics, and small molecule drugs. Furthermore, we will highlight the importance of multi-omics analysis techniques, chemical labeling methods, and *in vitro* models in elucidating the complex interactions within the gut-liver axis. By integrating these insights, this review seeks to offer valuable perspectives on the development of novel diagnostic biomarkers and therapeutic approaches for HCC.

## 2 The chemical properties and immunological impacts of microbial-derived components on liver cancer progression

The gut microbial produces metabolites such as secondary BAs, SCFAs, lactic acid, and ethanol, as well as microbial components like LPS from Gram-negative bacteria (Lagkouvardos et al., 2023; Yoshimoto et al., 2013). These metabolites and components play crucial roles in modulating hepatic immunity, metabolism, and tumor progression, *via* the gut-liver axis (Figure 1).

### 2.1 Lipopolysaccharide (LPS)

#### 2.1.1 LPS in HCC, immune activation and beyond

**FIGURE 2:**
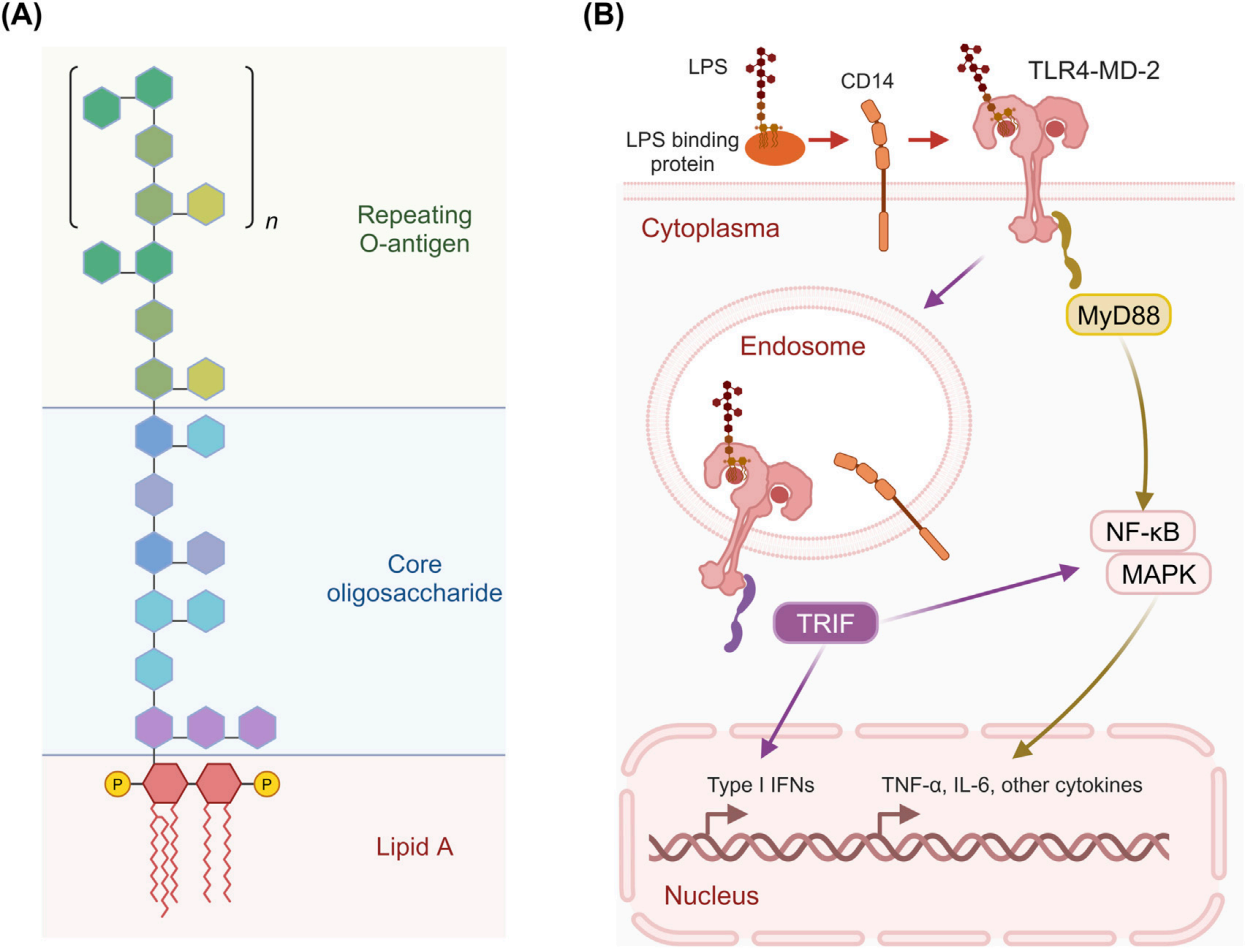
Structure of LPS and its activation of TLR4 signaling pathways. **(A)** LPS consists of three main parts: the repeating O-antigen, the core oligosaccharide, and Lipid A. The O-antigen is composed of repeating oligosaccharide units, while the core oligosaccharide links the O-antigen to Lipid A, which is embedded in the bacterial membrane and is responsible for the endotoxic activity of LPS. **(B)** LPS binds to LBP, which facilitates the transfer of LPS to CD14. LPS binds to LBP and is then transferred to CD14, which facilitates its interaction with the TLR4/MD-2 complex. This leads to the activation of two distinct signaling pathways: the MyD88-dependent pathway and the TRIF-dependent pathway. The recruitment of the adaptor protein MyD88 activates downstream signaling cascades involving NF-κB, MAPK, and other signaling molecules, resulting in the production of pro-inflammatory cytokines such as TNF-α and IL-6. The TRIF-dependent pathway leads to the production of Type I IFNs and other cytokines. Figure was created with BioRender.com.

LPS, a major component of the cell wall of Gram-negative bacteria and a well-known endotoxin, activates the host’s immune response by stimulating hepatic immune cells, particularly macrophages, *via* Toll-like receptor 4 (TLR4) and is considered a significant factor in promoting HCC progression by inducing liver inflammatory responses that lead to hepatocyte necrosis or apoptosis and subsequent liver injury (Singh et al., 2017; Luo et al., 2023). Specifically, LPS binds to LPS-binding protein (LBP), and the LBP-LPS complex is transferred to TLR4/MD-2 (myeloid differentiation factor 2) by cluster of differentiation 14 (CD14). The lipid A domain of LPS, which is relatively conserved, is responsible for most of its immunological activity and can be detected by the innate immune system at picomolar levels. MD-2 possesses a unique hydrophobic cavity where the acyl chain region of lipid A inserts into a specific binding pocket of MD-2, interacting with hydrophobic residues on both MD-2 and TLR4 to form a hexameric complex (Oblak and Jerala, 2015; Ohto et al., 2012). The binding of LPS induces TLR4 dimerization and recruits downstream signaling molecules, such as the adaptor protein myeloid differentiation factor 88 (MyD88) and TIR-domain-containing adapter inducing interferon-β (TRIF). MyD88 further activates downstream mitogen-activated protein kinase (MAPK) and nuclear factor-κB (NF-κB), leading to the release of pro-inflammatory mediators like tumor necrosis factor-α (TNF-α) and interleukin 6 (IL-6) (Figure 2B).

Beyond its direct role in immune activation *via* TLR4, LPS drives HCC progression through downstream signaling pathways activated by TLR4. Recent studies in mouse tumor models have shown that LPS promotes angiogenesis within tumor tissues by modulating the EGFR/IL8 and VEGFR/STAT3 signaling pathways, thereby enhancing tumor growth, proliferation, and metastasis (Kubo et al., 2024; Wang et al., 2019). Additionally, LPS upregulates the expression of HMGCR, LDLR, and SREBF2 while downregulating PCSK9 expression through the NF-κB pathway, leading to significant intracellular cholesterol accumulation and enhanced pro-inflammatory effects (He et al., 2017). In the context of HCC immunotherapy, LPS has also been reported to induce the expression of PD-1 and PD-L1 in mouse tumor tissues *via* the METTL14/MIR155HG pathway, providing new insights into HCC immunotherapy (Peng et al., 2022).

Despite the crucial role of TLR4 in HCC development, recent studies have shown that LPS can also act through non-TLR4 mechanisms (Dapito et al., 2012). A recent study has identified Galectin-3 as a potential LPS sensor, through which LPS can modulate glucose metabolism *via* the LPS/Galectin-3/Rag GTPases/Ragulator-mTORC1 axis, thereby participating in the regulation of HCC (Chen X. et al., 2022).

#### 2.1.2 Structural diversity of LPS and its immune implications

LPS typically consists of a hydrophobic lipid A domain, a more conserved nonrepeating core oligosaccharide, and an O-specific polysaccharide (or O-antigen) (Park and Lee, 2013) (Figure 2A). The lipid A domain comprises a phosphorylated di-glucosamine backbone, typically with phosphorylation at the 1 and 4′ positions, and four acyl groups linked at positions 2, 3, 2′, and 3’. Additionally, two secondary acyl chains are present on the distal glucosamine. The core oligosaccharides typically contain unusual carbohydrate residues, such as 3-deoxy-D-manno-oct-2-ulosonic acid residues, heptoses, and various hexoses, and are relatively conserved among bacterial species. The O-antigen is linked to the core oligosaccharide and typically consists of repeating oligosaccharides made of multiple sugars. It is the most diverse component of LPS, and some Gram-negative bacteria do not even synthesize this part.

The lipid A structure, while conserved at the species level, undergoes regulated modifications in response to varying environmental conditions, enabling bacteria to evade immune detection (Oblak and Jerala, 2015). For instance, *Yersinia pestis* and *Francisella tularensis* alter their lipid A composition based on temperature changes. Notably, lipid A from different bacterial species shows considerable structural diversity, particularly in terms of the number and length of acyl chains and modifications to the phosphate groups. Changes in the number and length of fatty acyl chains may affect their activity on TLR4, potentially acting as agonists or antagonists. The structural diversity of lipid A has been extensively studied, which is beneficial for the development of novel drugs. For example, by synthesizing lipid A analogs with specific structures, it is possible to design drugs that target specific bacteria. Here, we summarize the modifications of lipid A structures and their effects on immune recognition in different bacteria or environments that have been studied in recent years (Table 1).

**TABLE 1:** The lipid A structural diversity among bacteria.

Microbes	Acyl chain number and distribution	PO_4_	TLR4 activation	Characteristics	References
*F. nucleatum ATCC 51191*	6 (4:2)	1,4′bisP	ND	Mainly bis-phosphorylate hexa-acylated with 14:0, 14:0 (3-OH), and 16:0 (3-OH) chains in 4 + 2 symmetry; heterogeneous with penta- and tetra-acylated forms	Garcia-Vello et al. (2021)
*Escherichia coli*	6 (4:2)	1,4′bisP	++	Induces the production of pro-inflammatory cytokines and activation of immune cells such as macrophages and DCs	Maalej et al. (2019)
*Bacteroides vulgatus*	5 (2:3)	4′P	+	Restores homeostasis in experimental colitis models, low immunostimulatory potential	Di Lorenzo et al. (2020)
*Vibrio parahaemolyticus*	6 (4:2)	1,4′bisP	ND	Free lipid A and full-length LPS coexist	Huang et al. (2023)
*Alcaligenes faecalis*	6 (3:3)	1,4′bisP	ND	Maintains homeostatic immunological conditions within Peyer′s patches (PPs) by inducing IgA production through an IL-6-dependent mechanism	Shimoyama et al. (2021)
*Chlamydia trachomatis*	5 (2:3)	1P or 4′P	+	The lipid A fraction may be modified by phosphatidylethanolamine, a modification that could further reduce its immune activation capacity while increasing its resistance to host antimicrobial peptides	Yang et al. (2019)
*Proteus mirabilis*	6 (4:2)	1P or 4′-Ara4N-P	−	Can be modified by 4-amino-4-deoxy-L-arabinose (Ara4N), which usually occurs at the 4′-position phosphate group. This modification can increase the positive charge of lipid A, thereby interfering with the function of the cationic antimicrobial peptide and enhancing bacterial resistance	Yang et al. (2023a)
*Duganella aceris*	5 (2:3)	1Ara4N-P or 4′-Ara4N-P	+	Can be modified by 4-amino-4-deoxy-L-arabinose (Ara4N), which usually occurs at the 4′-position phosphate group. This modification can increase the positive charge of lipid A, thereby interfering with the function of the cationic antimicrobial peptide and enhancing bacterial resistance	Yang et al. (2023a)
*Pseudomonas lactucae*	6 (4:2)	1,4′Ara4N-bisP	−	Usually only a single Ara4N modification is performed, no double modifications were observed	Yang et al. (2023a)
*Massilia rubra*	6 (4:2)	1,4′Ara4N-bisP	ND	It is possible to have both Ara4N modifications at the 1-position and 4-position phosphate groups, a dual modification that is relatively rare in other bacteria. This dual modification may help bacteria survive and adapt in cold environments	Holochova et al. (2020)
*Rickettsia*	6 (4:2)	1,4′bisP	+	The longer fatty acid chains in most *Rickettsia* species could potentially enhance pro-inflammatory responses, while the shorter chains in R. rickettsii might modulate these responses differently	Guillotte et al. (2021)
*Achromobacter*	5 (2:3)	1P	+	Modifications of lipid A, such as GlcN addition and palmitoylation, increased the positive charge and hydrophobicity of lipid A, thereby reducing PmB binding and penetration and enhancing PmB resistance	MacDonald et al. (2023)
*Vibrio cholerae*	6 (4:2)	1,4′bisP	ND	Contains five different lipid A types, each of which differs in the number, length and chemical modification of acyl chains	Luna et al. (2021)
*Akkermansia muciniphila*	4 (2:2), 5 (2:3), 6 (4:2)	1P or 1,4P	TLR4: +, TLR2: ++	Capable of signaling through TLR2 and TLR4, but its activation of TLR2 is more pronounced	Garcia-Vello et al. (2024)

Abbreviations: ND, not determined; ++, strong activation of the MD-2/TLR4 complex; +, moderate activation; −, no activation or inhibition.

### 2.2 Bile acids (BAs)

#### 2.2.1 BAs: structure, synthesis, and metabolism

BAs are cholesterol-derived molecules that play essential roles in lipid digestion and metabolism. Structurally, BAs feature a steroid nucleus composed of four fused rings (three six-membered and one five-membered) and a side chain with multiple carbon atoms (Fuchs et al., 2025). The primary BAs in humans are cholic acid (CA) and chenodeoxycholic acid (CDCA), which differ in their hydroxyl (OH) and methyl (CH_3_) group arrangements. CA contains two hydroxyl groups (3α, 7α-OH) and a keto group (12-keto), while CDCA lacks the 7α-hydroxyl group.

BA synthesis primarily occurs in the liver through two main pathways: the classical pathway and the alternative pathway. The classical pathway, initiated by CYP7A1 and involving CYP8B1 and CYP27A1, generates CA and CDCA, accounting for about 90% of primary BAs in humans (Grant and DeMorrow, 2020). CYP8B1 determines the CA-to-CDCA ratio. The alternative pathway, involving CYP27A1 and CYP7B1, produces CDCA. In rodents, CDCA is rapidly converted into more hydrophilic α-muricholic acid (α-MCA) and β-muricholic acid (β-MCA) (Asgharpour et al., 2015). Primary BAs are conjugated with glycine or taurine in the liver to form glycocholic acid (GCA), taurocholic acid (TCA), glycochenodeoxycholic acid (GCDCA), and taurochenodeoxycholic acid (TCDCA) (Grant and DeMorrow, 2020). After synthesis, BAs are stored in the gallbladder and secreted into the small intestine *via* the biliary system postprandially. They facilitate lipid digestion and absorption, with most BAs being reabsorbed by intestinal cells and returned to the liver *via* the portal vein. A small fraction is excreted in feces (Di Ciaula et al., 2017).

**FIGURE 3:**
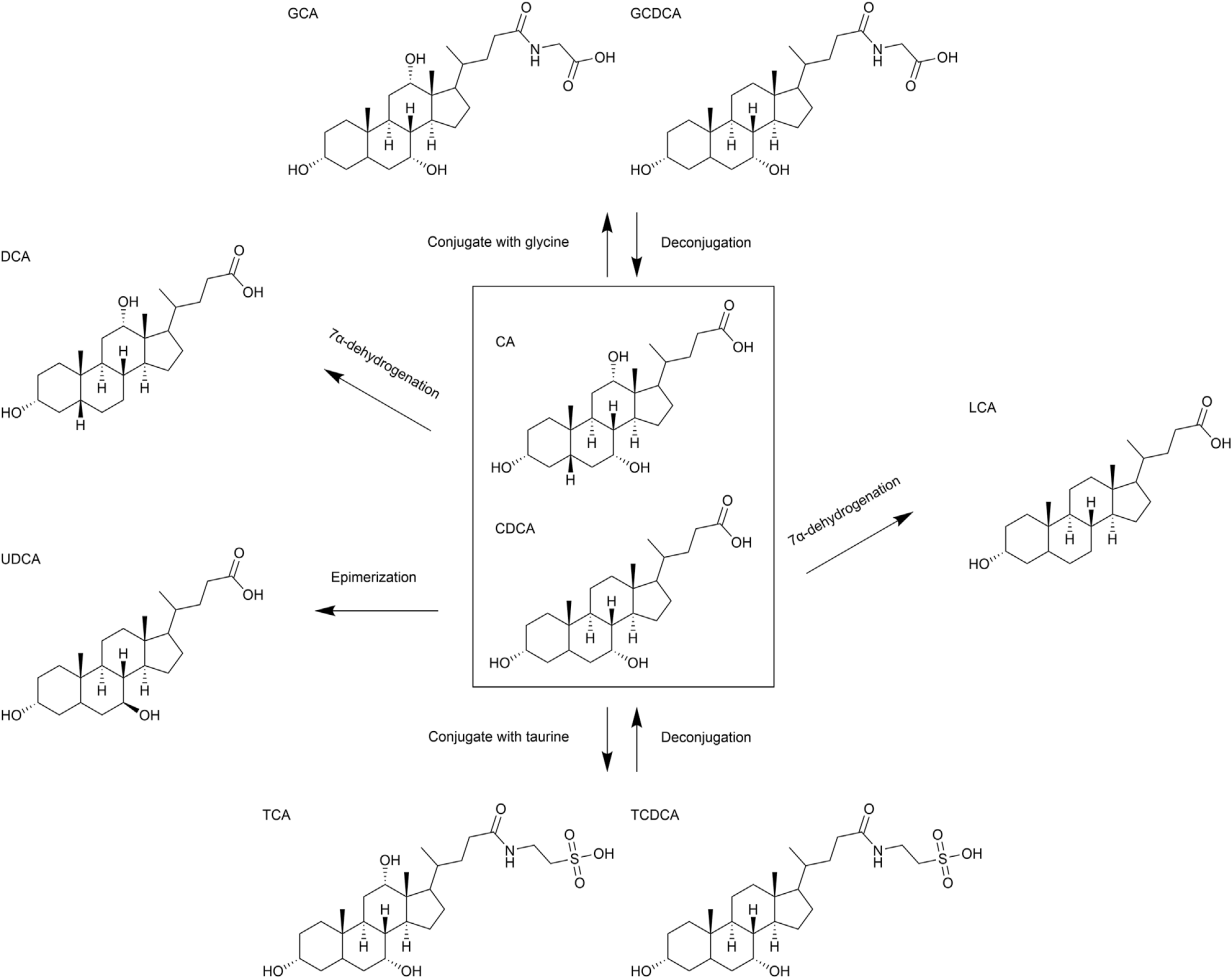
Schematic representation of structure and metabolism of bile acids (BAs). Major biochemical pathways involving the transformation of BAs, including conjugation with glycine and taurine, deconjugation, epimerization, 7α-dehydrogenation.

In the gut, BAs are metabolized by microbiota, primarily in the large intestine. Imbalances in gut microbiota can disrupt BA homeostasis, leading to conditions such as hepatitis, fibrosis, and HCC. Common microbial metabolic pathways include 7α-dehydrogenation, epimerization, and deconjugation (Figure 3). In addition to these pathways, BA metabolism also encompasses oxidation, esterification, desulfation, and further isomerization reactions, which collectively give rise to complex chemical structures within the gut (Choudhuri and Klaassen, 2022). Gut bacteria, such as *Bacteroides* and *Clostridium* species, convert primary BAs into secondary BAs, for example, deoxycholic acid (DCA) and lithocholic acid (LCA), *via* 7α-dehydroxylase, which removes the 7α-hydroxyl group (Fiorucci et al., 2021). *Eubacterium* and *Clostridium* species can catalyze the conversion of 7α-hydroxy BAs to 7β-hydroxy structures, for example, converting CDCA to ursodeoxycholic acid (UDCA), *via* 7-hydroxysteroid dehydrogenase (7-HSDH) (Doden et al., 2021). Additionally, gut bacteria such as *Bifidobacterium*, *Lactobacillus*, *Clostridium*, and *Enterococcus* express bile salt hydrolase (BSH), which deconjugates BAs by hydrolyzing glycine or taurine conjugates, facilitating further microbial metabolism (Doden et al., 2021).

#### 2.2.2 Impact of BAs on HCC progression and immune modulation

There is a close correlation between the metabolism of BAs in the gut and the progression of HCC as well as its response to immunotherapy. In the years preceding diagnosis, patients with HCC exhibit a significant increase in total BA concentrations, particularly an increased proportion of taurine-conjugated BAs (e.g., TCA and taurodeoxycholic acid), which are closely related to factors such as liver dysfunction and alterations in gut microbiota (Stepien et al., 2022). Another study found differences in the levels of primary and secondary BAs in plasma, liver, and gut in an N-nitrosodiethylamine-induced rat model. Levels of CDCA, CA, UDCA, and hyodeoxycholic acid in plasma can serve as early diagnostic biomarkers for HCC (Xing et al., 2023). Therefore, monitoring changes in the BA profile is of great significance for the early identification of high-risk populations for HCC, assessing disease progression, and guiding therapeutic strategies.

BAs have been shown to exert distinct effects on the development and treatment of HCC by selectively modulating the functions of specific hepatic immune cells such as macrophages and T cells, as well as non-immune cells like HSCs and liver sinusoidal endothelial cells (LSECs) (Table 2). For instance, TCA can promote the polarization of M2-like macrophages in the liver, thereby creating an immunosuppressive tumor microenvironment, which affects tumor immune evasion and growth (Sun et al., 2022). Studies have found that a high-fat diet or genetic obesity alters the gut microbiota, thereby increasing the level of the gut microbial metabolite DCA in the liver. This leads to HSCs senescence and the senescence-associated secretory phenotype, which secrete inflammatory cytokines, chemokines, proteases, and growth factors in the liver, promoting HCC progression (Yoshimoto et al., 2013; Lin et al., 2024). Additionally, certain BAs, such as UDCA, also exhibit potential for the treatment of liver diseases. For example, UDCA has been extensively studied for its efficacy in treating cholestasis and various other chronic liver conditions. UDCA can facilitate liver disease treatment by activating the farnesoid X receptor (FXR)/FGF-15 signaling pathway, regulating apoptosis and autophagy, enhancing intestinal barrier function, and reducing the toxicity of microbe-associated molecular patterns (MAMPs) (Mao et al., 2024). UDCA has also been reported to enhance CD8^+^ T cell function and inhibit tumor growth in mouse model of liver cancer by modulating bile acid composition and reducing ER stress in the tumor microenvironment (Varanasi et al., 2025). Primary BAs, such as CDCA and TCA, or secondary BAs, such as DCA and LCA, modulate the expression of the chemokine CXCL16 in LSECs. This alteration in CXCL16 expression subsequently influences the accumulation of NKT cells in the liver through CXCR6. Upon antigenic stimulation, NKT cells become activated and produce increased amounts of interferon-γ (IFN-γ), which is crucial for inhibiting liver tumor growth (Ma et al., 2018). Overall, these findings highlight the critical role of BAs in shaping the tumor microenvironment and influencing HCC progression through multiple pathways.

**TABLE 2:** Recent advances in the role of BAs in liver cancer.

Bile acids	Microbes	Potential mechanisms	Immune cells	Implication in HCC	References
TCA	Not involved	Promotes IL-4 induced M2-like macrophage polarization *via* FXR in the liver, creates an immunosuppressive tumor microenvironment.	Macrophages	Promotes the liver tumor immune evasion.	Sun et al. (2022)
	Not involved	Increases CXCL16 expression in LSECs, promote NKT cell accumulation in the liver.	NKT cells	Enhances antitumor immunity, and inhibit liver cancer growth.	Ma et al. (2018)
CDCA	Not involved	Increases CXCL16 expression in LSECs, promote NKT cell accumulation in the liver.	NKT cells	Enhances antitumor immunity, and inhibit liver cancer growth.	Ma et al. (2018)
DCA	Not identified	Suppresses T cell cytokine production dose-dependently	T cells	Not studied	Varanasi et al. (2025)
LCA	*Clostridium*	Decreases CXCL16 expression, reduce NKT cell accumulation in the liver.	NKT cells	Inhibits antitumor immunity, and promote liver cancer growth.	Ma et al. (2018)
	Not identified	Induces T cells dysfunction by promoting the expression of genes involved in T cell exhaustion (e.g., *Nr4a1*, *Nr4a2*) and upregulating ER stress-related genes (e.g., *Eif2ak3*, *Atf6*).	T cells	Impairs antitumor immunity and promote cancer progression.	Varanasi et al. (2025)
GCDCA	Not identified	Represses T cell survival dose-dependently.	T cells	Not studied	Varanasi et al. (2025)
TCDCA	Not identified	Induces ROS CD8^+^ T cells and represses T cell survival dose-dependently.	T cells	Not studied	Varanasi et al. (2025)
ω-MCA	*Clostridium*	Decreases CXCL16 expression, reduce NKT cell accumulation in the liver.	NKT cells	Inhibits antitumor immunity, and promote liver cancer growth.	Ma et al. (2018)
isoalloLCA	Not identified	Induces T cells dysfunction by promoting the expression of genes involved in T cell exhaustion (e.g., *Nr4a1*, *Nr4a2*) and upregulating ER stress-related genes (e.g., *Eif2ak3*, *Atf6*).	T cells	Impairs antitumor immunity and promote cancer progression.	Varanasi et al. (2025)
UDCA	Not identified	Enhances CD8^+^ T cell cytotoxicity (e.g., by increasing expression of *Gzmb* and cytokine secretion).	T cells	Improves T cell-mediated antitumor responses and reduces liver tumor growth.	Varanasi et al. (2025)
DCA	*Clostridium cluster XI and XIVa*	Induces senescence-associated secretory phenotype in HSCs.	Not studied	Promotes HCC development.	Yoshimoto et al. (2013)
GDCA	*Bifidobacteriales, Lactobacillales, Bacteroidales, Clostridiales*	Inhibits the proliferation and migration of HCC cells, and promotes the apoptosis of HCC cells.	Not studied	Inhibits the growth of HCC tumors.	Shen et al. (2022)
GCDCA	Not identified	Activates autophagy through the AMPK/mTOR signaling pathway.	Not studied	Promotes HCC invasion and metastasis.	Gao et al. (2019)

### 2.3 Short-chain fatty acids (SCFAs) in liver cancer

SCFAs, which are fermentation byproducts of dietary fiber and other nondigestible carbohydrates by gut microbiota, including acetate, propionate, and butyrate, serve as energy sources for intestinal epithelial cells and modulate the host’s immune response, energy metabolism, and overall gut health. SCFAs primarily exert their biological functions by interacting with G-protein-coupled receptors (GPCRs), particularly GPR41 and GPR43. These receptors play a crucial role in regulating glucose and lipid metabolism and inflammation in intestinal epithelial cells and immune cells. Notably, certain plant bioactive extracts, such as Eucommia bark and leaf extract (Wang Z. et al., 2023) or Arctigenin (Wang et al., 2024), can promote the production of SCFAs and activate the receptors GPR41 and GPR43 by modulating the gut microbiota, thereby alleviating lipid metabolic disorders and elevated blood glucose levels induced by a high-fat diet, as well as hepatic degenerative lesions.

SCFAs also modulate various immune cells, such as macrophages and type 3 innate lymphoid cells (ILC3s), through non-GPCR-dependent mechanisms. Recent studies have highlighted the significant implications of SCFAs in the development and progression of liver cancer (Table 3). For example, in mouse liver cancer models, acetate has been shown to decrease IL-17A levels by increasing Sox13 acetylation *via* histone deacetylase (HDAC) inhibition, thereby modulating the function of ILC3s and enhancing the efficacy of PD-1/PD-L1 checkpoint inhibition therapy (Hu C. et al., 2023). Similarly, in metabolic dysfunction-associated steatohepatitis (MASH) models, sodium butyrate induces macrophage polarization towards an M2 phenotype through HDAC inhibition, demonstrating anti-inflammatory properties (Sarkar et al., 2023).

**TABLE 3:** Recent advances in the role of SCFAs in liver cancer.

Acetate 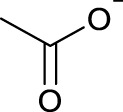		Propionate 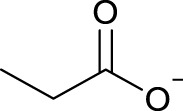		Butyrate 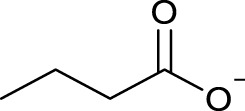	
SCFAs	Microbes	Potential mechanisms	Immune cells	Implication in HCC	References
Acetate	*Lactobacillus reuteri*	Reduces IL-17A+ ILC3 infiltration by regulating HDAC/Sox13/IL-17A signaling.	IL-17A+ ILC3s	Enhances anti-tumor immunity of PD-1/PD-L1 immune checkpoint inhibitors.	Hu et al. (2023a)
	*Bacteroides thetaiotaomicron*	Promotes the polarization of M1 macrophages and enhances the function of cytotoxic CD8^+^ T cells.	Macrophages, T cells	Inhibits the progression and recurrence of HCC.	Ma et al. (2024)
Butyrate	*Bifidobacteria, Lactobacilli*	Exerts anti-inflammatory effects by inhibiting HDACs, shifting M1 macrophage polarization towards an anti-inflammatory M2 phenotype, and inducing apoptosis in pro-inflammatory cells and cancer cells.	Macrophages	Reduces liver inflammation and fibrosis in MASH models, and lowers the risk of liver cancer development.	Sarkar et al. (2023)
	*Bacteroides caecimuris, Bacteroides xylanisolvens, Clostridium bolteae*	Induces the expansion of IL-10+ Tregs, suppress the immune response by inhibiting the activation and proliferation of cytotoxic CD8^+^ T cells, promoting an immunosuppressive environment.	T cells	Facilitates HCC development and progression.	Behary et al. (2021a)
Acetate	*Bifidobacterium pseudolongum*	Inhibits the IL-6/JAK1/STAT3 signaling pathway by activation of GPR43.	Not studied	Suppression of MASLD-HCC cell proliferation. Induction of cell apoptosis. Decreases intestinal permeability. Reduces tumor growth and progression.	Song et al. (2023)
	Not identified	Upregulates glutamine and UDP-GlcNAc levels and enhances protein O-GlcNAcylation in HCC.	Not studied	Promotes HCC progression	Zhou et al. (2023)
Butyrate	*Eubacterium rectale, Roseburia hominis, Bifidobacterium*	Activate the calcium signaling pathway, increase intracellular calcium levels, and promote ROS production.	Not studied	Inhibits cell proliferation, colony formation, and migration in HCC; enhances the efficacy of tyrosine kinase inhibitors, such as sorafenib.	Che et al. (2023)
	*Clostridia*	Not studied	Not studied	Prolonged exposure to butyrate promotes hepatocyte proliferation and liver fibrosis. Butyrate induces liver fibrosis and HCC tumorigenesis under certain conditions.	Singh et al. (2018)
	Not identified	Inhibits aerobic glycolysis (Warburg effect) in HCC cells by downregulating the expression of hexokinase 2 (HK2); suppresses the c-Myc signaling pathway, leading to decreased expression of anti-apoptotic protein Bcl-2 and increased expression of pro-apoptotic proteins (e.g., cleaved caspase 3 and 9).	Not studied	Promotes apoptosis and inhibits the proliferation of HCC cells, and enhances the anti-cancer effects of sorafenib.	Yu et al. (2022a)
	Not identified	Activates peroxisome proliferator-activated receptor alpha (PPARα) in LPS and fatty acid loaded cells. Reduces the secretion of pro-inflammatory cytokines such as TNF-α.	Not studied	Improves metabolic dysfunction and inflammation in hepatic cell, suggest potential protective effects against liver cancer.	Cook et al. (2023)
Propionate	Not identified	Activates peroxisome proliferator-activated receptor alpha (PPARα) in LPS and fatty acid loaded cells. Reduces the secretion of pro-inflammatory cytokines such as TNF-α.	Not studied	Improves metabolic dysfunction and inflammation in hepatic cell, suggest potential protective effects against liver cancer.	Cook et al. (2023)
Acetate, Butyrate, Propionate (not specifically characterized)	Not identified	Suppresses Ras activity by promoting DAB2 expression. Downregulates PI3K, VEGF, transforming growth factor-beta, interferon signaling, and inflammation associated pathways.	Not studied	Inhibits cancer cell proliferation; delays the pathogenesis of HBV-associated HCC.	McBrearty et al. (2021)

Additionally, SCFAs have been implicated in upregulating tumor suppressors such as Disabled homolog 2 (DAB2), inhibiting Ras signaling, and blocking the progression from chronic viral liver disease to HCC (McBrearty et al., 2021). Butyrate’s ability to regulate intracellular calcium homeostasis and reactive oxygen species (ROS) further underscores its potential to enhance the antitumor activity of sorafenib against HCC (Che et al., 2023). The potential detrimental effects of SCFAs in HCC are also noteworthy (Singh et al., 2018; Zhou et al., 2023; Behary et al., 2021a). Long-term intake of large amounts of soluble dietary fiber (such as inulin and fructooligosaccharides) may lead to the production of excessive SCFAs *via* gut microbiota fermentation. In certain cases, this has been shown to induce cholestasis, liver inflammation, and liver cancer in mice (Singh et al., 2018; Zhou et al., 2023; Behary et al., 2021a). This indicates that under specific conditions, the overproduction of SCFAs may have negative impacts on liver health.

### 2.4 Other components

In addition to the aforementioned substances, recent studies have explored the effects of other bacterial components or metabolites on liver cancer progression. Lipoteichoic acid, a major component of the cell wall of Gram-positive bacteria, can accumulate in the liver due to obesity. In obese-induced HCC mouse models and MASH-related HCC patients, lipoteichoic acid accumulation activates caspase-11 to cleave gasdermin D, which in turn facilitates the release of IL-33 and IL-1β from stellate cells, thereby promoting cancer progression (Yamagishi et al., 2022).

Specific gut bacteria, such as *Enterococcus faecium*, enhance the proportion of IFN-γ+CD8^+^ T cells in the tumor microenvironment by inducing IL-12 and IFN-γ secretion. The exopolysaccharides produced by *E. faecium* further promote IFN-γ secretion by these T cells, synergistically inducing ferroptosis in HCC cells with sorafenib and enhancing its therapeutic efficacy in advanced HCC (Yu et al., 2024). In HCC patients, the microbiota’s functional profile shifts from carbohydrate metabolism to amino acid metabolism (Behary et al., 2021b). Compared to healthy controls, concentrations of trimethylamine-related metabolites are elevated, along with increases in p-cresol glucuronide, indole-lactic acid, 5-hydroxyindoleacetic acid, and 4-hydroxyphenyllactic acid (Banerjee et al., 2024). Trimethylamine N-oxide, which is a metabolite of trimethylamine produced in the liver, exacerbates chronic hepatic inflammation by inducing vascular endothelial cell damage, increasing M1 macrophages, and reducing M2 macrophages, thereby promoting MASLD progression (Nian et al., 2024; Zhang X. et al., 2023).

Overall, these findings highlight the diverse roles of bacterial components and metabolites in liver cancer progression, emphasizing the need for further research to elucidate their mechanisms and potential therapeutic applications.

## 3 Designing of therapeutics using the gut-liver axis

### 3.1 Recent advancements in targeted therapies for gut microbiota modulation

The “core microbiota” in the human intestine comprises genera such as *Candida*, *Saccharomyces*, *Penicillium*, and *Aspergillus* (Nash et al., 2017). In healthy adults, the gut microbiota is predominantly composed of *Bacteroidetes* and *Firmicutes*, with smaller proportions of *Proteobacteria*, *Actinobacteria*, and *Verrucomicrobia* (Mariat et al., 2009). Although bacterial diversity expands rapidly in infancy, it stabilizes in adulthood but continues to evolve due to geographical, nutritional, and lifestyle influences (Cheng et al., 2016).

Recent studies have highlighted alterations in the gut microbiota during various stages of liver cancer development, characterized by a reduction in beneficial bacteria and an increase in potentially harmful bacteria, leading to dysbiosis. For instance, in elderly HCC patients aged 60–80 years, researchers observed significant decreases in beneficial genera such as *Blautia*, *Fusicatenibacter*, *Anaerostipes*, and others, while harmful genera like *Escherichia-Shigella*, *Cronobacter*, and *Megasphaera* were significantly more abundant (Zhang W. et al., 2023). Similar trends were seen in patients with alcoholic liver diseases and HBV-related HCC, where specific bacterial taxa were either depleted or enriched (Lu et al., 2016; Temraz et al., 2021; Ganesan et al., 2024; Huang et al., 2020).

These alterations in gut microbiota and their metabolites play crucial roles in regulating the initiation and progression of liver diseases. They also influence the efficacy and toxicity of cancer therapies through various mechanisms. Given the significant impact of the gut microbiota on disease outcomes, recent advancements in targeted therapies for gut microbiota modulation have shown promise. Current methods include FMT, phage therapy, antibiotics, probiotics, dietary interventions, and small molecules (Woo et al., 2023). These therapies can regulate host inflammation and immune status, thereby affecting cancer treatment outcomes. This summary outlines examples of the latest research progress on study of therapies for the treatment of HCC and other liver diseases (Table 4).

**TABLE 4:** Examples of recent progress in gut microbiota-based liver disease therapies studies.

Intervention	Models	Liver diseases	Potential mechanisms of action	References
FMT	CCl4-HCC mouse model	HCC	Increases metabolites SCFAs and acetate, and reduces IL-17A production.	Hu et al. (2023a)
FMT	High-fat, high-cholesterol diet mouse model	MASLD-HCC	Adjust the metabolites of the recipient mice, such as TCDCA and 3-indolepropionic acid (IPA)	Zhang et al. (2021)
FMT	Human	HCC	Enhances the antitumor effects of radiotherapy by modulating the gut microbiome and activating the Cgas/STING signaling pathway.	Li et al. (2022a)
FMT	Mouse model	Immune Agonist Antibodies induced liver disease	Regulates MyD88-dependent signaling pathways.	Blake et al. (2021)
FMT	Mouse model	Autoimmune hepatitis (AIH)	Inhibits the TLR4/11-MyD88 signaling pathway, it effectively restores the mouse gut microbiota and improves the imbalance between TFR cells and TFH cells in the spleen.	Ma et al. (2023), Liang et al. (2021)
Phage therapy	Mouse model	MASLD	Targeting *Klebsiella pneumoniae* (HiAlc Kpn)	Gan et al. (2023)
Phage therapy	Mouse model	Alcoholic hepatitis	Targeting *Enterococcus faecalis*	Duan et al. (2019)
Probiotics	Mouse model	MASLD-HCC	The Bifidobacterium pseudolongum metabolite acetate, acting on the hepatic GPR43 receptor, it subsequently inhibits the IL-6/JAK1/STAT3 signaling pathway.	Song et al. (2023)
Probiotics	Mouse model	T2DM+HCC	Improve the diversity of the gut microbiota, reduces BAs in the serum, and regulates the MMP9 and NOTCH1 signaling pathways	Chen et al. (2023a)
Probiotics	Mouse model	HCC	It may enhance the effectiveness of immunotherapy by affecting BA metabolism and increasing serum TUDCA levels, thereby increasing the proportion of CD8^+^ T cells in the tumor microenvironment	Lan et al. (2024)
Probiotics	High-fat diet mouse model	Metabolic associated fatty liver disease	Activate the Acly/Nrf2/NF-κB signaling pathway and inhibit the differentiation of macrophages into pro-inflammatory M1 macrophages.	Shao et al. (2022)
Probiotics	Mouse model, human	AIH	Regulate Tfh cell responses through CD103+ DCs and the MyD88/NF-κB signaling pathway.	Ma et al. (2022)
Probiotics	Mouse model	Impaired liver-resident NK cells	Restore the function of liver NK Cells through the metabolite butyrate.	Tian et al. (2023a)
Probiotics	Mouse model	Alcoholic liver disease	Enhance the activity of NK cells.	Eom et al. (2023)
Small molecular	Mouse model	MASLD	OCA increases the abundance of beneficial gut microbiota, thereby upregulating the alternative BA synthesis pathway (especially Cyp7b1), reducing the levels of hydrophobic BAs and increasing the levels of serum-conjugated BAs.	Liu et al. (2023), Lee et al. (2023)
Small molecular	Mouse model	MASH	Dual agonists target the GLP-1/GLP-2 receptors.	Kim et al. (2022)
Small molecular	Mouse model	MASH	GW9662 inhibits PPARγ/CD36 signaling.	Xiao et al. (2023)
Small molecular	Mouse model	HCC	2,5-dimethylcelecoxib enhances the antitumor functions of natural killer (NK) cells and T cells by modulating the gastrointestinal microbiota-AMPK-mTOR axis.	Pan et al. (2023)
Small molecular	Mouse model	HCC	Aloe emodin increases relative abundance of *Bacteroides* and *Lactobacillus*, primary BAs and NKT cells.	Deng et al. (2024)
Small molecular	High-fat, high-cholesterol diet mouse model	MASLD	Nobiletin improves the balance of gut microbiota and metabolism of myristoleic acid.	Li et al. (2023)

#### 3.1.1 Fecal microbiota transplantation (FMT)

Despite the underlying mechanisms are not fully elucidated, FMT has been shown to restore gut microbiota balance, thereby influencing the tumor immune microenvironment and enhancing antitumor treatment effects (Qin et al., 2022), particularly by enhancing the antitumor effects of immune checkpoint inhibitors.

In addition to its role in cancer therapy, FMT has shown preliminary efficacy in treating MASLD, MASH, and cirrhosis by restoring intestinal barrier function and re-regulating dysbiosis. For example, a recent study in an experimental autoimmune hepatitis (AIH) mouse model found that gut microbiota dysbiosis exacerbated liver damage and imbalanced Tfr/Tfh cells (Ma et al., 2023; Liang et al., 2021). FMT effectively restored gut microbiota dysbiosis caused by antibiotic treatment in AIH mice by inhibiting the TLR4/MyD88 signaling pathway, improved the imbalance between splenic Tfr and Tfh cells, and thereby controlled hepatitis and liver damage progression.

However, the specific mechanisms by which FMT affects liver cancer development through gut microbiota regulation are still not fully understood. Further preclinical studies are needed to elucidate these mechanisms, and well-designed clinical trials are required to verify the efficacy and safety of FMT in treating liver diseases and enhancing cancer immunotherapy.

#### 3.1.2 Phage therapy

Phage therapy, characterized by its high specificity, can modulate the gut microbiota by eliminating specific pathogenic bacteria, thereby improving gut health. Although current research is still in its early stages, studies have indicated that phage-mediated gut microbiota modulation may help suppress liver cancer progression. For instance, research suggests that high-alcohol-producing *Klebsiella pneumoniae* (HiAlc Kpn) is associated with MASLD. Targeted phage therapy against HiAlc Kpn can regulate hepatic lipid metabolism and inflammation, reduce the expression of genes related to fatty acid synthesis, and mitigate disease progression (Gan et al., 2023). In patients with alcoholic hepatitis, fecal levels of *E. faecalis* are significantly higher than in non-drinkers, and the expression levels of hemolytic *Enterococcus faecalis* are positively correlated with increased mortality. Targeted phage therapy against hemolytic *E. faecalis* has been shown to effectively ameliorate gut microbiota imbalances in mice with alcoholic hepatitis (Duan et al., 2019). These findings suggest that phage therapy holds promise for treating liver diseases by specifically targeting pathogenic bacteria and restoring microbial balance.

#### 3.1.3 Probiotics

Probiotics, such as *Bifidobacterium* and *Lactobacillus* species, enhance immunity by regulating gut microbiota balance. In HCC patients receiving PD-1 monoclonal antibody immunotherapy, *Akkermansia muciniphila* improves treatment efficacy by modulating BA metabolism and increasing serum TUDCA levels, thereby increasing CD8^+^ T cells infiltration in the tumor microenvironment (Lan et al., 2024). While *Clostridium butyricum* alone does not alleviate metabolic-associated fatty liver disease in mice, its combination with soluble dietary fiber significantly increases gut microbiota diversity, interferes with lipid synthesis, inhibits macrophage differentiation towards M1, and exerts anti-inflammatory effects, thereby inhibiting disease progression (Shao et al., 2022). Clinical data analysis shows that Tfh, Tfr, and Treg cell levels, as well as the Tfr-Tfh index, are higher in HCC patients than in those with chronic hepatitis and healthy controls, with a high Tfr-Tfh index significantly associated with HCC recurrence (Wang B. et al., 2020). *Lactobacillus* enhances the suppressive effect of prednisone on Tfh responses *via* CD103^+^ DCs and the MyD88/NF-κB pathway, improving therapeutic outcomes in AIH patients and animal models (Ma et al., 2022). Probiotics, including *Phocaeicola dorei*, *Lactobacillus helveticus*, and *C. butyricum*, can also enhance NK cell activity by improving gut microbiota balance (Tian P. et al., 2023; Eom et al., 2023). Early antibiotic treatment disrupts IL-18/IL-18R signaling in Kupffer cells and hepatocytes, inhibiting the functional maturation of NK cells in the liver, but this can be restored by supplementing with specific butyrate-producing bacteria such as *C. butyricum* (Tian P. et al., 2023). The acetate produced by *Bifidobacterium pseudolongum* inhibits the IL-6/JAK1/STAT3 signaling cascade *via* the GPR43, blocking tumor cell cycle progression and enhancing apoptosis, which may attenuate the progression of MASLD to HCC (Song et al., 2023).

In addition to their immunomodulatory effects, probiotics regulate host metabolic functions. For example, *Lactobacillus gasseri LA39* enhances primary BA synthesis in the liver by regulating proteins like CYP27A1 and promotes secondary BA levels in the gut, thereby modulating BA metabolism along the liver-gut axis (Hu J. et al., 2023). *Lactobacillus brevis* ameliorates the pathological transition from type 2 diabetes to liver cancer in mouse models by modulating serum BA concentrations and regulating matrix metalloproteinase-9 (MMP9) and NOTCH1 signaling pathways (Chen S. et al., 2023). These findings highlight the multifaceted roles of probiotics in modulating gut microbiota, enhancing immune responses, and regulating metabolic functions, thereby offering potential therapeutic benefits for liver diseases and cancer.

#### 3.1.4 Small molecules

Small molecules that target specific receptors and modulate gut microbiota balance have emerged as promising therapeutic agents for liver diseases. For example, dual agonists targeting the GLP-1/GLP-2 receptors significantly improve gut microbiota composition in MASH model mice, enhance intestinal barrier tight junctions, and thus alleviate disease phenotypes (Kim et al., 2022). Similarly, the PPARγ antagonist GW9662 increases beneficial bacteria (e.g., *Dubosiella* and *Lactobacillus*) and reduces harmful bacteria (e.g., Helicobacteraceae, *Desulfovibriaceae*, and *Rickenaceae*), thereby improving gut microbiota disorders and alleviating MASH progression (Xiao et al., 2023).

Natural product compounds also contribute to the modulation of gut microbiota and the treatment of liver diseases. For instance, 2,5-dimethylcelecoxib, a celecoxib derivative, regulates gut microbiota (e.g., *Bacteroides acidifaciens*, *Odoribacter laneus*, and *Odoribacter splanchnicus*) and activates the AMPK/mTOR signaling pathway in leukocytes. This activation enhances the antitumor capabilities of NK cells and T cells, inhibits immune cell exhaustion, and effectively suppresses liver cancer growth, improving HCC prognosis in mouse models (Pan et al., 2023). Nobiletin, a natural flavonoid compound from citrus fruits, reverses gut microbiota dysbiosis in MASLD mouse models and alleviates MASLD symptoms by improving myristic acid metabolism (Li et al., 2023). Aloe emodin exhibits anti-HCC activity by increasing the relative abundance of *Bacteroides* and *Lactobacillus*, promoting BSH accumulation, increasing primary BA content, and triggering IFN-γ production in NKT cells in the liver (Deng et al., 2024).

### 3.2 Immune-based therapies progress focusing on TLR4 and FXR as representative targets

Immune-based therapies targeting the gut-liver axis hold significant promise for the treatment of HCC. Strategies such as FMT and probiotics have been shown to modulate the gut microbiota and enhance the efficacy of immune checkpoint inhibitors. Additionally, the use of small molecule drugs that target specific immune-regulatory pathways, such as TLR4 and FXR, has shown potential in liver diseases studies (Supplementary Table 1).

TLR4 promotes inflammatory responses and liver fibrosis progression by activating downstream signaling pathways, such as the MyD88/NF-κB axis, and plays a significant role in various liver diseases, including MASLD, hepatic fibrosis, cirrhosis, and HCC (Tang et al., 2023). Inhibiting TLR4 signaling triggered by PAMPs like LPS to reduce inflammation and slow fibrosis development may represent a promising therapeutic opportunity. Several natural products have been identified as TLR4 inhibitors. For example, studies in mouse liver cancer models induced by dimethyl nitrosamine and CCl4 have shown that ginsenoside Rk3 can significantly inhibit LPS-induced TLR4 signaling, reduce inflammatory cytokine expression, and thus alleviate liver fibrosis (Qu et al., 2021). Echinacea purpurea polysaccharide (EPP) and red rice seed coat extract have also been shown to inhibit the growth of mouse liver cancer cells by modulating the TLR4-mediated NF-κB pathway (Jing et al., 2024; Chen Y. et al., 2023). Nimbolide, another plant extract, has been shown in mouse liver cancer models to alleviate gut microbiota imbalance by increasing the relative abundance of *Bifidobacterium* and *Lactobacillus*, enhance intestinal barrier integrity by upregulating tight junction-related protein ZO-1 expression, and reduce inflammation by downregulating the TLR4-mediated NF-κB pathway activity, thereby inhibiting tumorigenesis and development (Ram et al., 2022).

BAs exert their effects through various receptors, such as FXR and Takeda G protein-coupled receptor 5 (TGR5), which play crucial roles in modulating liver cancer progression. FXR, a nuclear receptor primarily expressed in the intestines, liver, and other tissues, regulates BAs, glucose, and lipid metabolism. Activation of FXR by BAs can modulate downstream signaling pathways, such as NF-κB, thereby reducing the release of inflammatory cytokines and exerts anti-inflammatory effects. Recent research has explored the potential of FXR agonists in treating liver cancer and related conditions. For example, obeticholic acid (OCA), an FXR agonist, has been shown to ameliorate MASLD symptoms by modulating gut microbiota composition, enhancing the abundance of beneficial intestinal microbes such as *A. muciniphila*, *Bifidobacterium spp*., and *Bacteroides spp*., and regulating BA synthesis and serum levels (Liu et al., 2023; Lee et al., 2023; Gou et al., 2022). Natural products have also been studied for their ability to regulate FXR function. For instance, celastrol, a well-known natural product, has been found in rat liver cancer models to reduce the relative abundance of *Bacteroides fragilis*, increase glycine binding to BAs, and alleviate HCC by regulating the (FXR/RXRα)/mTOR axis (Zeng et al., 2023).

Recent advancements in research methodologies, such as molecular docking and molecular dynamics simulations, combined with AI-driven screening technologies, hold great potential for revolutionizing the discovery of small molecule drugs targeting receptors like TLR4 and FXR. Researchers have used molecular docking and molecular dynamics simulations to study the tetramer formation of the TLR4/MD2 complex and the key residues involved, which aids in discovering small molecule antagonists that can block TLR4 signaling (Tafazzol and Duan, 2019). Based on structural similarity to OCA, 109 FDA-approved drugs were selected from the PubChem database. Molecular docking combined with GROMACS software for molecular dynamics simulations was employed to evaluate the stability of drug-protein complexes and their ADMET (absorption, distribution, metabolism, excretion, and toxicity) properties, ultimately revealing that Montelukast and Alvimopan are more druggable for further development (Jose et al., 2023). In a recent study, a team from Tsinghua University used AI technology, employing next-generation virtual screening platforms HelixVS and HelixDock, to identify a novel pyrazolo [1,5-a]pyrimidine derivative, TH023, which targets the TLR4-TLR4 homodimerization interface and inhibits TLR4 signaling (Jiang et al., 2024).

## 4 Multi-omics analysis technique and *in vitro* models in deciphering the interactions of the gut-liver axis

### 4.1 Utilization of multi-omics analysis technique to understand and characterize the gut-liver axis

The gut-liver axis represents a complex, bidirectional communication network. With the advancement of multi-omics analysis techniques, such as genomics, proteomics, and metabolomics, gut microbes and their metabolites can be identified with greater precision. This has significantly deepened our understanding of their roles in the pathogenesis of HCC. Multi-omics analysis methods can be applied to the diagnosis, treatment, and prognosis prediction of cancer. By comparing the small molecule metabolic profiles of healthy individuals with those of patients with liver disease, researchers can identify specific metabolic pathways or metabolites associated with disease progression, thereby revealing changes in the metabolic activities of the gut microbiota and their impact on liver diseases. For instance, in several studies, researchers have conducted microbiome and transcriptome analyses on fecal samples, tumor tissues, and adjacent non-tumor tissues from a large number of HBV-associated HCC patients. These studies found that various bacteria, including *Bacteroides*, *Clostridium XIVa*, *Dialister*, *Veillonella*, and *Streptococcus pneumoniae*, were more abundant in HCC patients. These bacteria may influence the occurrence, progression, and prognosis of HCC by regulating metabolites such as serum BAs, acetate, glutamate, and arachidonic acid (Huang et al., 2020; Zheng et al., 2023).

Bioinformatic tools can assist in the analysis of omics data. For example, in the study of the gut-liver axis, MetaboAnalyst can be used to process and analyze metabolomics data, including the processing of liquid chromatography-mass spectrometry (LC-MS) data, functional annotation, statistical analysis, and integration of multi-omics data. Differential expression analysis of metabolites using MetaboAnalyst can help researchers identify metabolic biomarkers related to the gut-liver axis and further explore the role of these metabolites in the pathogenesis of liver diseases (Hu et al., 2021).

By integrating bioinformatics methods to conduct in-depth analyses of multi-omics results, we can more comprehensively elucidate the pathogenesis and provide new insights into the mechanisms of HCC (Shi et al., 2023; Cui et al., 2023). In a recent study focusing on the mRNA expression and clinical follow-up information of HCC patients, the TCGA dataset and regression analysis were utilized to identify 18 BAs metabolism-related genes significantly associated with HCC prognosis. A risk model was developed to predict the prognosis of liver cancer patients and their response to immunotherapy (Shi et al., 2023). Long non-coding RNAs (lncRNAs) data have also garnered attention in constructing predictive models for the treatment response and prognosis of HCC patients (Cui et al., 2023).

Multi-omics analysis also provides a more comprehensive understanding of the molecular mechanisms within the gut-liver axis, elucidating how specific microbes contribute to liver diseases. A study conducted in a murine model of Type 2 Diabetes Mellitus (T2DM) and HCC employed 16S rRNA sequencing and gas chromatography-mass spectrometry (GC-MS) to analyze the regulatory effects of gut microbiota, particularly *Actinomycetes*, as well as microbial metabolites, notably total BAs, following treatment with *L. brevis* (Chen S. et al., 2023). Furthermore, the research delved deeper through transcriptomic analysis to investigate the impact of the treatment on gene expression, revealing that MMP9 and NOTCH1 signaling pathways may play pivotal roles in disease alleviation.

Alternatively, multi-omics analysis techniques have also made substantial contributions to clarifying the mechanisms through which some traditional Chinese medicines or natural compounds exert their therapeutic effects (Deng et al., 2024; Jing et al., 2024; Chen Y. et al., 2023; Zeng et al., 2023). For example, recent studies have utilized 16S rRNA gene sequencing and untargeted metabolomics to investigate the intervention mechanism of EPP on HCC (Jing et al., 2024). The results indicate that EPP modulates the gut microbiota, particularly by increasing the abundance of gut microorganisms that produce propionate and butyrate (such as *Coprococcus*, *Clostridium*, and *Roseburia*). This enhances the expression of intestinal tight junction proteins, thereby repairing the intestinal barrier, controlling the leakage of LPS, and inhibiting the TLR4/NF-κB signaling pathway. In another study investigating the therapeutic effects of the traditional Chinese medicine “Xiayuxue decoction” (XYXD) on HCC, researchers employed ultra-performance liquid chromatography coupled with quadrupole time-of-flight mass spectrometry (UPLC-Q-TOF-MS) to identify the major active components in XYXD (Deng et al., 2024). Among these, aloe emodin was found to be the most effective. Furthermore, metagenomic and metabolomic analyses revealed that the active components promote the production of BSH by increasing the abundance of *Bacteroides* and *Lactobacillus*. BSH, in turn, regulates the metabolism of primary BAs, which triggers the production of IFN-γ by NKT cells in the liver, thereby exerting an immunotherapeutic effect against HCC.

### 4.2 Utilization of *in vitro* models to understand and characterize the gut-liver axis

Organoid models, which simulate the three-dimensional structure and function of tissues, offer a more physiologically relevant experimental platform for studying intestinal and liver diseases (Gunther et al., 2022; Shek et al., 2021). For example, liver organoids have been employed to elucidate the pathophysiological mechanisms of liver diseases such MASLD and MASH (He et al., 2023). To better mimic the complex interactions between the intestine and liver, researchers have developed cell-based Caco-2/HepG2 co-culture models to simulate the metabolic processes of sugars in the human digestive system and their impact on liver health (van Laar et al., 2022).

In recent years, advances in microfluidic technology have led to significant progress in developing *in vitro* models of the gut-liver axis. The Gut-liver chip, also known as Organoids/organs-on-a-chip, represents one of the important breakthroughs in this field. This technology employs microfluidic techniques to construct co-culture organoid models, recapitulating the physiological structure and function of the intestine and liver (Wu et al., 2023; Guo et al., 2023). For instance, Choe et al. used microfluidic technology to co-culture intestinal epithelial cells and liver cells, simulating intestinal absorption and liver metabolism processes and reproducing first-pass metabolism dynamics (Choe et al., 2017). The Gut-liver chip also allows researchers to investigate how microbial metabolites affect hepatocyte functions, including BA synthesis, glucose and lipid metabolism (Choe et al., 2017), and the secretion of albumin and urea (Kang et al., 2023). Additionally, this technology can be utilized to study the interactions of the gut-liver axis under specific disease conditions, such as fatty liver disease and acute liver failure (Yang J. et al., 2023; Liu et al., 2024).

However, despite the great potential of these new *in vitro* models in gut-liver axis research, several challenges remain. For example, accurately simulating the complex intercellular signaling and microenvironment remains a critical issue (Kang et al., 2023). Compared to organoids derived from healthy tissues, those from HCC and cholangiocarcinoma exhibit downregulated expression of pattern recognition receptors such as TLRs, which is inconsistent with *in vivo* observations (Shek et al., 2021). Therefore, there is a need to develop *in vitro* models that more closely reflect *in vivo* characteristics for drug screening and therapeutic applications.

### 4.3 Application of chemical labeling and analytical techniques used in characterize the gut-liver axis

The gut microbiota and its metabolites play a crucial role in the gut-liver axis, highlighting the urgent need for comprehensive and in-depth studies on their composition and distribution. The application of novel chemical labeling and analytical techniques provides strong support for this research endeavor.

**FIGURE 4:**
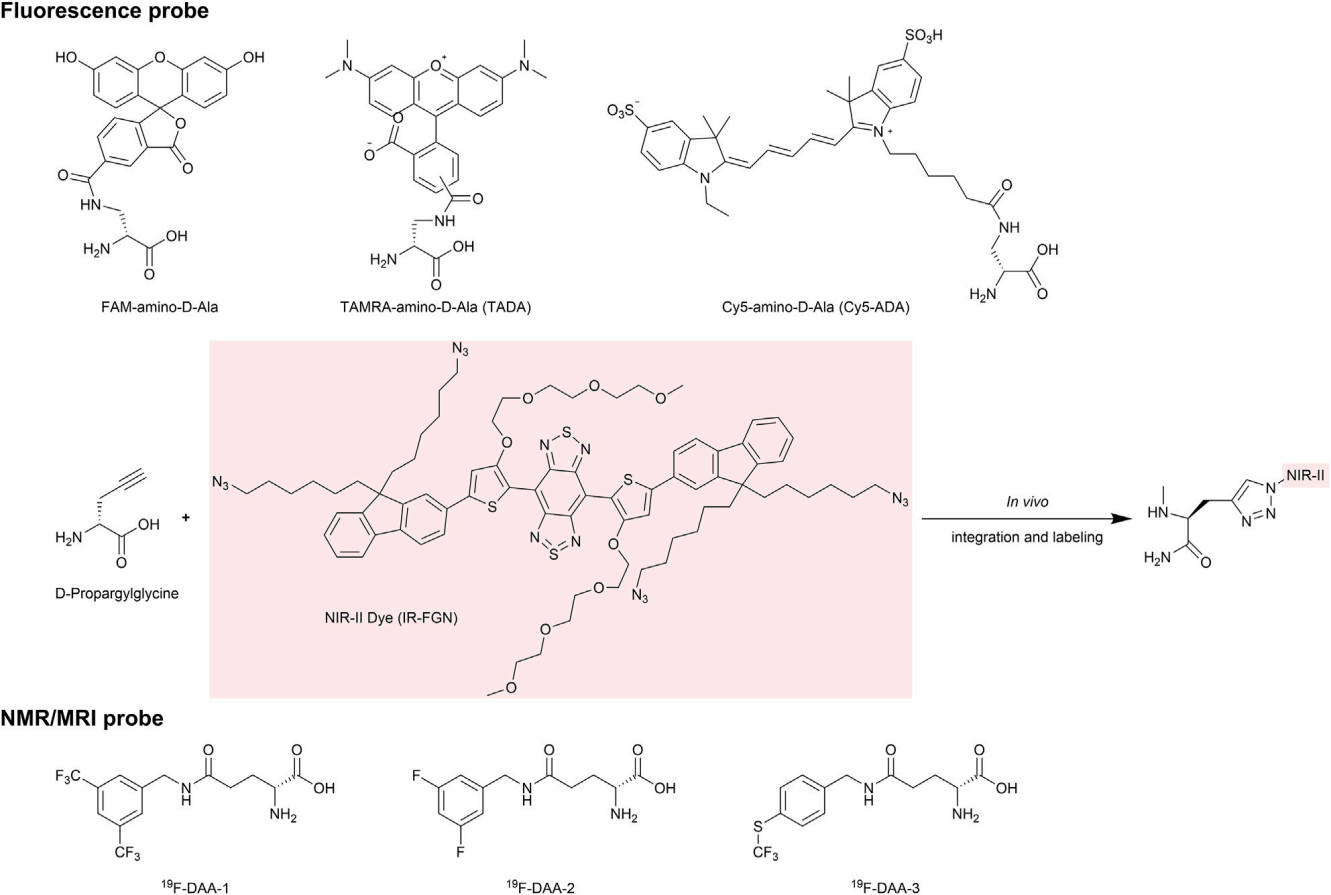
Representative D-amino acid (DAA) based chemical probes. The chemical structures of probes used for in vivo imaging are shown, including D-Ala probes labeled with fluorophores such as FAM, TAMRA, and Cy5. Additionally, the D-Propargylglycine probe is designed for in vivo labeling with NIR-II dyes via an azide group. By replacing the fluorescent label with a ^19^F modification, the probe can be used for higher sensitivity and deeper penetration in NMR imaging.

Chen et al. employed fluorescence labeling to observe the encapsulation and release of probiotics in the gut (Chen QW. et al., 2022), thereby aiding in the optimization of probiotic therapies. They labeled different probiotics with fluorescein coumarin and Cy5, respectively, and examined the distribution of these labeled probiotics within microspheres using fluorescence microscopy. This approach confirmed successful encapsulation and simulated their release in a gut environment. In recent years, a series of D-amino acid (DAA) based chemical probes have been developed to study the metabolism and activity of gut microbiota (Lin et al., 2020a; Lin et al., 2020b; Chen D. et al., 2023; Wang W. et al., 2020) (Figure 4). These probes, constructed by linking fluorescent groups, isotopes, and near-infrared (NIR-II) groups to DAAs, are incorporated into peptidoglycan structures during bacterial metabolism. This enables non-invasive and non-perturbative bacterial labeling and allows for the visualization of metabolic activities, such as the growth and division of different gut bacteria, *in vivo*.

Chemical labeling techniques, such as isotope labeling, can enhance the sensitivity of metabolomics analysis (Yuan et al., 2018; Kaur et al., 2023; Lin et al., 2021; Garg et al., 2018; Conway et al., 2019; Lin et al., 2023). In a recent study, researchers developed a method for analyzing fecal metabolites through integrated chemical isotope labeling combined with liquid chromatography-mass spectrometry (CIL-LC-MS). Different chemical isotope labeling reagents were used to label metabolites containing carboxyl, carbonyl, amino, and thiol groups, respectively. Using specific mass spectrometry methods, a systematic analysis of the metabolites in mouse feces was conducted, revealing significant differences in 211 metabolites between model mice and wild-type (WT) mice (Yuan et al., 2018).

The use of specific metabolite-binding probes for the separation and analysis of analytes can also help enhance analytical sensitivity. For instance, bicyclobutane, serving as a selective probe, is conjugated to magnetic beads *via* diethylenetriamine (Kaur et al., 2023). This probe-magnetic bead system labels and separates thiol metabolites, improving the sensitivity and specificity of thiol metabolite detection by ultra-high-performance liquid chromatography-tandem mass spectrometry (UPLC-MS/MS). Lin et al. also employed a novel selective probe for the absolute quantification of SCFAs at low nanomolar concentrations (Lin et al., 2023). The probe consists of a magnetic bead substrate, a selective reaction group (N-methylbenzylamine), and a bioorthogonal cleavable group (*p*-nitrocinnamyloxy-carbonyl, Noc). SCFAs react with the selective reactive group through a nucleophilic addition-elimination mechanism to form stable covalent bonds, allowing for their separation. Coupled with a^13^C_6_ isotope-labeled internal standard of SCFAs and UHPLC-MS, this method achieves high-sensitivity, high-reproducibility quantitative analysis of SCFAs. The limit of detection (LOD) and limit of quantification (LOQ) of the assay were very low, 1 nM and 10 nM, respectively.

Chemical labeling techniques can also be employed to enhance the capabilities of proteomic analysis (Han et al., 2025). Gut microbes express a variety of BSH, which regulate the composition of the host’s BA pool. Researchers designed activity-based probes based on the cores of different BAs, such as CA, CDCA, DCA, and LCA. These probes incorporate a fluoromethylketone (FMK) warhead to covalently label the active site of BSH and an azide group as a click chemistry handle for subsequent detection and analysis. This enables the identification and assessment of BSH substrate specificity towards various BA substrates. The probes enrich and identify BSHs with different substrate specificities through Cu-catalyzed azide-alkyne cycloaddition (CuAAC) click chemistry. This approach provides a new perspective for developing precise microbial community intervention strategies or novel drugs to regulate BA metabolism.

## 5 Summary and prospects

The gut-liver axis is a complex, bidirectional communication network involving chemical and biological interactions. This review summarizes the intricate relationship between the gut-liver axis and HCC, highlighting the significant roles of gut microbiota and its derived molecules, such as LPS, BAs, and SCFAs, in disease progression and treatment.

Understanding the compositional, chemical, and immunological changes underlying the gut-liver axis is crucial, as these alterations can serve as potential diagnostic biomarkers and therapeutic targets. The application of multi-omics analysis techniques, chemical labeling and analytical technologies, coupled with bioinformatics tools and *in vitro* models, has greatly enhanced our understanding of the molecular mechanisms of the gut-liver axis in HCC and facilitated the discovery of potential biomarkers. These novel research findings bring hope to therapeutic strategies targeting liver diseases *via* the gut-liver axis. Approaches such as FMT, phage therapy, probiotics, and small molecule drugs aim to restore the balance of the gut microbiota, enhance immune responses, and mitigate the deleterious effects of dysregulated microbial metabolites. Natural products or small molecule drugs identified through high-throughput screening and chemical structural optimization have also shown potential in reducing inflammation, fibrosis, and tumor growth by interfering with key signaling pathways involved in HCC development.

In conclusion, the gut-liver axis represents a promising research and therapeutic avenue for HCC. However, the complex interaction mechanisms among the gut microbiota, host immune system, and hepatocytes are not yet fully elucidated and require further investigation. Existing studies mostly rely on animal models, which may not fully reflect the complexity of human diseases. Future research should focus on elucidating the complex interactions among the gut microbiota, microbial metabolites, immune system, and liver diseases, with a particular emphasis on identifying novel immunotherapeutic targets. Given individual differences, more targeted intervention measures, such as biological therapies and small molecule therapies targeting specific bacteria or metabolic pathways, should be formulated. Additionally, larger-scale clinical trials are essential to validate the efficacy and safety of therapies targeting the gut-liver axis and to explore potential synergistic effects when combined with existing HCC treatments. The continuous investigation of the chemical interactions and biological effects of gut microbiota-derived components will undoubtedly provide valuable insights and lay the foundation for the development of innovative therapeutic strategies to combat liver cancer.
