# Supplementary material for: Insights into the gut-liver axis: mechanisms and emerging therapies in hepatocellular carcinoma
Source: Front Pharmacol. 2025 May 19;16:1595853. doi: 10.3389/fphar.2025.1595853 (PMC12127201; doi:10.3389/fphar.2025.1595853)
Supplement: Supplementary file 1 [file Table1.docx]

Supplementary Table 1. Recent progress of TLR4 and FXR compounds studies.

| **Target receptor** | **Categories** | **Sources** | **Smole molecular name** | **Structures** | **Potential mechanisms of action** | **References** |
| --- | --- | --- | --- | --- | --- | --- |
| TLR4/MD-2 | Antagonist | Synthetic | TAK-242 (Resatorvid) |  | Binds directly to TLR4, preventing the formation of the TLR4/MD-2 complex and inhibiting downstream signaling pathways (e.g., NF-κB). | Plunk et al. (2020) |
|  | Inactive Prodrug | Synthetic | Tak-242 Prodrug 2 (p-Nitrobenzyl Carbamate Prodrug) |  | Inactive until converted to Tak-242 by nitroreductase. Once activated, it binds to TLR4 to inhibit signaling. | Plunk et al. (2020) |
|  | Inactive Prodrug | Synthetic | Tak-242 Prodrug 3 (p-Nitrobenzyl Alkylated Prodrug) |  | Inactive until converted to Tak-242 by nitroreductase. Once activated, it binds to TLR4 to inhibit signaling. | Plunk et al. (2020) |
|  | Inactive Prodrug | Synthetic | Tak-242 Prodrug 4 (Propargyl Alkylated Prodrug) |  | Inactive until converted to Tak-242 by Pd0 catalysis. Once activated, it binds to TLR4 to inhibit signaling. | Plunk et al. (2020) |
|  | Inactive Prodrug | Synthetic | Tak-242 Prodrug 5 (Propargyl Carbamate Prodrug) |  | Inactive until converted to Tak-242 by Pd0 catalysis. Once activated, it binds to TLR4 to inhibit signaling. | Plunk et al. (2020) |
|  | Antagonist | Synthetic | Mygalin |  | Binds to the MD-2 protein, a coreceptor of TLR4, and sequesters LPS, preventing the activation of TLR4 signaling pathways. It also interacts with LPS directly, neutralizing its activity. | Espinoza-Culupu et al. (2020) |
| TLR4/MD-2 | Antagonist | Synthetic | Z-20 |  | Exerts its anti-inflammatory effects by directly binding to the TLR4/MD2 complex and inhibiting downstream ERK signaling pathways, thereby reducing LPS-induced organ injury and inflammation. | Zeng et al. (2020) |
|  | Antagonist | Synthetic | AVR-48 / AVR-25 |  | Interacts with TLR4 to modulate macrophages towards an anti-inflammatory phenotype. | Shah et al. (2021) |
|  | Antagonist | Synthetic | Hit 94 |  | May bind to the active site or allosteric sites of the TLR4/MD-2 complex, preventing the recognition of LPS and subsequent signaling cascade activation. | Zhang et al. (2023c) |
|  | Antagonist | Natural product | Curcumin |  | Binds to the MD-2 protein, competing with LPS for the binding site and preventing TLR4 activation. | Espinoza-Culupu et al. (2020), Li et al. (2024a) |
|  | Antagonist | Natural product | Geniposide |  | Directly binds to TLR4 and inhibits the TLR4/MyD88 signaling pathway, prevents the activation of downstream signaling molecules, including MyD88, p38 MAPK, NF-κB, and the transcription factors STAT3 and Sp1.  Inhibits tumor angiogenesis by suppressing endothelial cell migration and tube formation. | Zhang et al. (2020a) |
|  | Antagonist | Natural product | Saponarin |  | Interacts directly with the TLR4/MD2 complex, binding to the hydrophobic pocket of MD2 where LPS typically binds.  Inhibits the formation of the TLR4/MyD88 signaling complex, thereby blocking downstream pro-inflammatory signaling pathways, including MAPK (ERK1/2, JNK, p38) and NF-κB. | Yu et al. (2022b) |
| TLR4/MD-2 | Antagonist | Natural product | Ginsenoside Rk3 |  | Interacts with TLR4 via hydrogen bonds at positions Tyr102, Ser118, and Ser120, inhibiting the TLR4 signaling pathway.  Downregulates the expression of TLR4 and its downstream signaling molecules, such as NF-κB and MyD88, thereby reducing inflammation and liver injury. | Qu et al. (2021) |
|  | Antagonist | Endogenous | Tripeptide Arg-Lys-His (RKH) |  | Directly binds to TLR4 and competes with LPS for binding to the TLR4/MD2 complex | Xie et al. (2023) |
|  | Antagonist (human), Agonist (mouse) | Endogenous | C16-sulfatide |  | Binds to MD-2, inducing receptor dimerization in mice.  Acts as an antagonist in human cells by competing with lipid A.  (Longer FA chain length reduces binding affinity and activation compared to C12-sulfatide.) | Su et al. (2021) |
|  | Inhibitor | Synthetic | Compound 3l |  | Binds to the LPS binding site of TLR4/MD-2, disrupting TLR4/MD-2 heterodimerization and TLR4 homodimerization. Blocks the NF-κB/MAPK signaling pathway by preventing TLR4 activation, thereby reducing the expression of inflammatory mediators (e.g., NO, TNF-α, IL-1β). | Liu et al. (2021) |
|  | Inhibitor | Natural product | Crocin (CROC) |  | Exhibits molecular antagonism by interacting with TLR4 and TGF-βR1, as shown by molecular docking studies. Protects against cisplatin-induced hepatotoxicity by reducing TLR4 and TGF-β signaling, enhancing BAMBI expression, and mitigating oxidative stress. | Khedr et al. (2020) |
|  | Inhibitor | Natural product | Scutellarein (SCU) |  | Directly binds to TLR4, disrupting the interaction between TLR4 and TRAF6.  Inhibits the canonical NF-κB pathway by preventing the phosphorylation and nuclear translocation of NF-κB p65. | Shi et al. (2025) |
| TLR4/MD-2 | Inhibitor | Natural product | Hydroxysafflower yellow A (HSYA) |  | Suppresses the TLR4/NF-κB signaling pathway by potentially inhibiting the activation of TLR4 receptors and downstream NF-κB transcription factors. | Feng et al. (2022) |
| FXR | Agonist | Endogenous | CDCA |  | Strongly activates FXR, leading to downstream effects such as increased expression of fibroblast growth factor 15 (FGF15) in the ileum.  Improves glucose metabolism in PCOS model mice, reducing fasting blood glucose and mean blood glucose levels. | Yang et al. (2021) |
|  | Agonist | Endogenous | Agmatine |  | Promotes polycystic ovary syndrome like symptoms by activating the FXR pathway, which inhibits GLP-1 secretion by L cells, leading to insulin resistance and ovarian dysfunction. | Yun et al. (2024) |
|  | Agonist | Endogenous | Compound F4/5 |  | Attenuates colitis by selectively activating FXR, which likely modulates inflammatory pathways and gut homeostasis. | Huo et al. (2022) |
|  | Agonist | Synthetic | GW4064 |  | Binds to and activates the FXR, restores FXR signaling in iron-overloaded mice, mitigating iron-induced hepatotoxicity. | Xiong et al. (2022) |
|  | Agonist | Synthetic | OCA |  | Alleviates the histological and biochemical features of MASH without cirrhosis | Rinella et al. (2022) |
| FXR | Agonist | Synthetic | HEC96719 |  | Binds to FXR with higher potency, showing a 150-fold improvement compared to GW4064. | Cao et al. (2022) |
|  | Agonist | Synthetic | INT-767 |  | Binds to FXR and TGR5, activating downstream pathways involved in lipid metabolism and inflammation.  Significantly reduces intestinal ischemia reperfusion injury induced damage by decreasing inflammation, preserving intestinal barrier integrity, and reducing endotoxin translocation. | Anfuso et al. (2020), Canovai et al. (2023) |
|  | Agonist | Synthetic | Nidufexor (LMB763) |  | Binds to the FXR ligand-binding domain, fitting into hydrophobic and polar pockets. The core oxygen forms a weak hydrogen bond with Y373, and the terminal amide oxygen interacts with R335.  Acts as a partial FXR agonist, reduces hepatic steatosis, inflammation, and fibrosis in murine MASH models. | Chianelli et al. (2020) |
|  | Agonist | Synthetic | Compound 3a |  | FXR Agonist, leukemia inhibitory factor receptor (LIFR) Antagonist.  Protects against liver fibrosis and inflammation by binding to the ligand-binding domain and stabilizing the receptor complex to activate downstream signaling. | Rapacciuolo et al. (2024) |
|  | Agonist | Synthetic | Compound 5/11 |  | Dual FXR antagonist and PXR agonist.  Reverts the expression of FXR target genes involved in BA excretion and reduces the production of proinflammatory factors in HepG2 cells. | Finamore et al. (2023) |
|  | Agonist | Synthetic | LH10 |  | Binds to FXR, forming hydrogen bonds with key amino acids like HIS298 to activate downstream pathways, thereby regulating lipid metabolism, inflammation, oxidative stress, and fibrosis and improving cholestasis, acute liver injury, and MASH pathology in mouse models. | Huang et al. (2024) |
| FXR | Agonist | Synthetic | HPG1860 |  | Acts as a full FXR agonist by binding to the FXR ligand-binding domain, stabilizing its active conformation, and modulating key gene expressions involved in BA metabolism, inflammation, fibrosis, and lipid/glucose homeostasis, thereby showing potential to reduce liver inflammation and fibrosis in rodent models of MASH. | Mo et al. (2023) |
|  | Agonist | Synthetic | Compound 4/5 |    | Provides protection against acetaminophen-induced hepatotoxicity by activating FXR. | Cai et al. (2022) |
|  | Agonist | Synthetic | Cilofexor |  | Exerts antisteatotic and antifibrotic effects by modulating BA, lipid, and cholesterol metabolism through FXR activation | Hollenback et al. (2024) |
|  | Agonist | Synthetic | Compound 10a (optimized from XJ034) |  | Forms an additional hydrogen bond with TYR383 in the FXR receptor, enhancing its transactivation activity.  Improves hyperlipidemia, hepatic steatosis, and insulin resistance in diet-induced obese mice. | Qin et al. (2023) |
|  | Agonist | Synthetic | Compound 3a |  | Reduces liver steatosis, inflammation, and fibrosis by activating the FXR pathway. | Zhang et al. (2023d) |
|  | Agonist | Synthetic | BMS-986339 |  | Targets FXR to regulate BA homeostasis, potentially reducing inflammation and fibrosis in MASH. | Nara et al. (2022) |
| FXR | Agonist | Synthetic | Compound 19 |  | Alleviates histological features of fatty liver, including steatosis, lobular inflammation, ballooning, and fibrosis, through FXR activation in MASH model mice. | Wang et al. (2023b) |
|  | Agonist | Synthetic | TC-100 (3α, 7α, 11β-Trihydroxy-6α-ethyl-5β-cholan-24-oic Acid) |  | Activates FXR and induces the expression of Fibroblast Growth Factor 15/19 (FGF15/19), which inhibits hepatic BA synthesis via the repression of CYP7A1. | Marzano et al. (2022) |
|  | Agonist | Synthetic | Compound 33 |  | Activates FXR to modulate hepatic-intestinal circulation, intestinal metabolism, immunity, and microbial regulation, thereby promoting repair of colonic epithelium in a DSS-induced acute enteritis model. | Li et al. (2024b) |
|  | Agonist | Synthetic | SU5 |  | Regulates lipid metabolism and triglyceride metabolism, improve fatty liver by activating FXR. | Wen et al. (2022) |
|  | Agonist | Natural product | Auraptene |  | Regulates lipid metabolism and triglyceride metabolism, improve fatty liver by activating FXR. | Wen et al. (2022) |
|  | Agonist | Natural product | Gypenosides (Gyps) |  | Directly activates FXR and upregulates its target genes such as small heterodimer partner (SHP).  Regulates lipid metabolism by downregulating SREBP1, FASN, and SCD1, and upregulating CPT1A and LPL.  Improves high-fat diet-induced MASH by reducing hepatic triglyceride content. | Li et al. (2022b) |
|  | Agonist | Natural product | Swertiamarin (SW) |  | Alleviates cholestasis by reversing changes in BA metabolism and related proteins (e.g., CYP7A1, NTCP, BSEP, MRP2) via FXR signaling. | Shi et al. (2022) |
| FXR | Agonist | Natural product | Ginsenoside Rc |  | Binds to FXR, enhancing its transcriptional activity and upregulating downstream target genes such as SHP and Bile Salt Export Pump (BSEP).  Alleviates acetaminophen-induced hepatotoxicity by reducing oxidative stress, inflammation, and apoptosis. | Zhong et al. (2022) |
|  | Agonist | Natural product | Alisol B 23-acetate (ABA) |  | Binds with residues R331 and S332 in the ligand-binding domain of the FXR, activating FXR, which induces the expression of downstream target genes such as SHP1, MRP2, BSEP, and AQP2.  Reduces renal inflammation, apoptosis, and oxidative stress in ischemia-reperfusion injury (IRI)-induced acute kidney injury (AKI). | Luan et al. (2021) |
|  | Agonist | Natural product | Berberine (BBR) |  | Significantly lowers blood glucose levels by modulating FXR signaling and inhibiting hepatic gluconeogenesis. | Sun et al. (2021) |
|  | Agonist | Natural product | 20*S*-protopanaxatriol |  | Binds to the FXR to increase its expression.  Inhibits ECM deposition, reduces proinflammatory cytokines (e.g., caspase 1, IL-1β, IL1R1, IL-6), and suppresses HSCs activation and inflammation via FXR activation. | Song et al. (2020) |
|  | Agonist | Natural product | Hesperidin |  | Makes hydrophobic contacts with amino acids in the FXR ligand-binding pocket.  Regulates FXR-target genes such as BSEP, multidrug resistance-associated protein 2 (MRP2), and Na^+^-taurocholate cotransporting polypeptide (NTCP). | Zhang et al. (2020b) |
| FXR | Agonist | Natural product | Isotschimgine |  | Exerts anti-obesity and anti-hepatic steatosis effects by altering the expression levels of FXR downstream genes, lipid synthesis, and energy metabolism genes in high-fat diet-induced obese mice liver. | Li et al. (2020) |
|  | Agonist | Natural product | Schaftoside |  | Protects mice from acetaminophen-induced hepatotoxicity by activating FXR, which in turn induces phase II and III detoxifying enzymes and modulates the generation of pro- and anti-inflammatory eicosanoids, thereby reducing oxidative stress and inflammation in the liver. | Liu et al. (2020) |
|  | Agonist | Natural product | Cafestol |  | Binds to these receptors and upregulates genes involved in fatty acid β-oxidation (e.g., ech-1.1) and energy expenditure (e.g., tub-1).  Reduces fat accumulation by increasing fat oxidation and energy expenditure. | Farias-Pereira et al. (2020) |
|  | Agonist | Natural product | kaempferol-7-O-rhamnoside |  | Binds stably to FXR through hydrophobic interactions with residues such as HIS294, MET265, ILE335, and LEU348, and hydrogen bonds with SER342. | Liu et al. (2022) |
|  | Agonist | Natural product | 2-Oxokolavenol |  | Binds to the FXR LBD, forming hydrogen bonds with key residues. Induces recruitment of coactivators (SRC1-2 and SRC2-3) and release of corepressors (NCoR2), leading to FXR transcriptional activation.  Alleviates acetaminophen induced hepatocyte damage by activating FXR, which regulates downstream target genes involved in liver metabolism and inflammation. | Guo et al. (2022) |
|  | Agonist | Natural product | Licraside |  | Activates FXR and leads to the upregulation of proteins like SHP and BSEP, which help regulate BA homeostasis by reducing BA synthesis, uptake, and promoting BA excretion. | Xi et al. (2023) |
| FXR | Agonist | Natural product | Compound 27 |  | Ameliorates α-naphthyl isothiocyanate induced cholestasis by modulating FXR activity and related signaling pathways (e.g., upregulating SHP and BSEP expression, and downregulating CYP7A1 and NTCP expression.), resulting in improved liver histopathology and reduced biochemical markers associated with liver damage. | Yao et al. (2024) |
|  | Antagonist | Endogenous | TUDCA |  | Improves glucose metabolism and insulin sensitivity. | Therdtatha et al. (2021) |
|  | Antagonist | Synthetic | Compound 9a (9,11-seco-cholesterol derivatives) |  | Inhibits FXR in the liver, reducing the expression of Fxr and Shp, which may affect BA levels and metabolism. | Zhou et al. (2022) |
|  | Antagonist | Synthetic | FLG249 |  | Regulates FXR downstream genes (e.g., Fgf15, Asbt, Shp) in the ileum, potentially affecting metabolic pathways. | Teno et al. (2021) |
|  | Antagonist | Synthetic | Glycine-β-muricholic acid (Gly-MCA) |  | Reduces hepatic lipid accumulation, inflammation, and fibrosis in MASH models by inhibiting the intestinal FXR/ceramide axis. | Jiang et al. (2022) |
|  | Antagonist | Natural product | Celastrol |  | Enhances gastrointestinal injury by inhibiting FXR signaling, which synergizes with triptolide to activate the JNK pathway and induce intestinal bleeding.  Alleviates HCC proliferation by regulating the Bacteroides fragilis/GUDCA/(FXR/RXRα)/ mTOR axis. | Dai et al. (2023), Zeng et al. (2023) |
| FXR | Antagonist | Natural product | Curcumin |  | FXR antagonist, TGR5 agonist.  Enhances GLP-1 release through L-cell expansion, mediated by the gut microbiota-BAs-TGR5/FXR axis. | Tian et al. (2023b) |
